# Analgesics use and ESRD in younger age: a case-control study

**DOI:** 10.1186/1471-2369-8-15

**Published:** 2007-12-05

**Authors:** Fokke J van der Woude, Lothar AJ Heinemann, Helmut Graf, Michael Lewis, Sabine Moehner, Anita Assmann, Doerthe Kühl-Habich

**Affiliations:** 1 Nephrology, 5. Med. Klinik, Klinikum Heidelberg-Mannheim, Theodor-Kutzer-Ufer 1-3, 68167 Mannheim, Germany; 2 Centre for Epidemiology & Health Research Berlin, Invalidenstr. 115, 10115 Berlin, Germany; 3 Neprologie, Krankenanstalt der Stadt Wien, Rudolfstifting, 3. Med. Abteilung, Juchgasse 25, 1030 Wien, Austria; 4 EPES Epidemiology, Pharmacoepidemiology and Systems Research GmbH, Wulfstr. 8, 12165 Berlin, Germany

## Abstract

**Background:**

An ad hoc peer-review committee was jointly appointed by Drug Authorities and Industry in Germany, Austria and Switzerland in 1999/2000 to review the evidence for a causal relation between phenacetin-free analgesics and nephropathy. The committee found the evidence as inconclusive and requested a new case-control study of adequate design.

**Methods:**

We performed a population-based case-control study with incident cases of end-stage renal disease (ESRD) under the age of 50 years and four age and sex-matched neighborhood controls in 170 dialysis centers (153 in Germany, and 17 in Austria) from January 1, 2001 to December 31, 2004. Data on lifetime medical history, risk factors, treatment, job exposure and intake of analgesics were obtained in a standardized face-to-face interview using memory aids to enhance accuracy. Study design, study performance, analysis plan, and study report were approved by an independent international advisory committee and by the Drug Authorities involved. Unconditional logistic regression analyses were performed.

**Results:**

The analysis included 907 cases and 3,622 controls who had never used phenacetin-containing analgesics in their lifetime. The use of high cumulative lifetime dose (3^rd ^tertile) of analgesics in the period up to five years before dialysis was not associated with later ESRD. Adjusted odds ratios with 95% confidence intervals were 0.8 (0.7 – 1.0) and 1.0 (0.8 – 1.3) for ever- compared with no or low use and high use compared with low use, respectively. The same results were found for all analgesics and for mono-, and combination preparations with and without caffeine. No increased risk was shown in analyses stratifying for dose and duration. Dose-response analyses showed that analgesic use was not associated with an increased risk for ESRD up to 3.5 kg cumulative lifetime dose (98 % of the cases with ESRD). While the large subgroup of users with a lifetime dose up to 0.5 kg (278 cases and 1365 controls) showed a significantly decreased risk, a tiny subgroup of extreme users with over 3.5 kg lifetime use (19 cases and 11 controls) showed a significant risk increase. The detailed evaluation of 22 cases and 19 controls with over 2.5 kg lifetime use recommended by the regulatory advisors showed an impressive excess of other conditions than analgesics triggering the evolution of ESRD in cases compared with controls.

**Conclusion:**

We found no clinically meaningful evidence for an increased risk of ESRD associated with use of phenacetin-free analgesics in single or combined formulation. The apparent risk increase shown in a small subgroup with extreme lifetime dose of analgesics is most likely an indirect, non-causal association. This hypothesis, however, cannot be confirmed or refuted within our case-control study. Overall, our results lend support to the mounting evidence that phenacetin-free analgesics do not induce ESRD and that the notion of "analgesic nephropathy" needs to be re-evaluated.

## Background

The publications by Spühler *et al. *[1], Dubach *et al. *[2] and Zollinger [3] five decades ago on the relationship between kidney damage and phenacetin ultimately led to the recognition of a causal relation between the two. This was supported by the experience in the Swedish factory Husqvarna [4]. Many epidemiological studies and review papers contributed to this view. In the 1990ies, many nephrologists by extension suspected that non-phenacetin analgesic combinations, especially those containing paracetamol and aspirin (plus caffeine) might also cause end-stage renal disease (ESRD; cf. reviews [5-7]), and introduced the term "analgesic nephropathy." Since data were scarce, this hypothesis engendered some controversy [8,9], and ultimately led the regulatory authorities of Germany, Austria and Switzerland to initiate a scientific re-evaluation. A peer review committee of scientists, jointly selected by the regulatory authorities and the pharmaceutical industry, was asked to critically review the data on the relationship between non-phenacetin combined analgesics and nephropathy on the basis of a systematic literature review. The committee's three main conclusions were that (1) existing studies show no justified suspicion of any increased risk of ESRD for phenacetin-free analgesic combinations, (2) there is insufficient evidence to associate non-phenacetin combined analgesics with nephropathy and (3) new studies should be done to provide appropriate data to resolve the issue [10].

In their initial deliberations on such a study, the Scientific Board recommended that the outcome of interest be ESRD (defined as incident dialysis), and that the exposure of interest be all phenacetin-free analgesics, including NSAIDs), regardless of the formulation. The board was aware that the analysis would address several subgroups, including analgesics co-formulated with caffeine. The design efforts resulted in a detailed, published study protocol, confirmed and approved by the regulatory authorities [11].

The primary objective of this study was to determine the association between the lifetime use of all phenacetin-free analgesics and the occurrence of ESRD. ESRD risk was to be evaluated for several mono substances ("Monos"), fixed combination analgesics ("Combis"), by cumulative lifetime dose, duration, and dose by duration of use of phenacetin-free analgesics. This paper presents the main results of the international case-control study on the association of phenacetin-free analgesics and ESRD.

## Methods

The study was designed as a population-based case-control study to investigate the risk of phenacetin-free analgesics with regard to the occurrence of ESRD in Germany and Austria between January, 2001 and December, 2004. The study protocol was reviewed and agreed to by the Scientific Advisory Committee, was accepted by the regulatory authorities of Germany, Austria, and Switzerland, and was reviewed and approved by the Kidney Foundation of Germany. It was supported by the three Nephrological Societies in Germany and the Nephrological Association of Austria, published in BMC Nephrology [11], shown on an open-access website [12], registered as clinical study in the FDA website (Protocol Registration Receipt NCT00302835) [13], and approved as a study protocol by the Lancet.

Cases were recruited from 170 dialysis centers and were defined as patients with end-stage renal disease (ESRD) newly admitted to a chronic dialysis program (incident dialysis) and aged up to 50 years. Rapid case ascertainment systems were used to identify all eligible cases. Cases were excluded if they had acute or recurrent renal failure, were out of age range, died before the interview could be done, were in poor physical or mental condition, or refused informed consent to participate in the study. Four neighborhood controls were randomly selected from the same geographic regions as the cases and were matched by age (5-year age group) and sex. Cases and controls documented their willingness to participate in the study by signing an informed consent form.

All study participants completed a standardized in-person interview, during which a trained interviewer collected information on lifetime use of analgesics documented by brand name as well as details and dates of lifetime use, on co-morbidity (renal, circulatory, metabolic, psychiatric, other medical conditions, headache or other painful conditions), treatment history (treatments with potential relation to outcome and risk factors), job exposure (occupation & industrial branch; exposure in selected occupational categories; exposure to groups of certain chemicals and minerals), and other demographic data such as age, sex, area of residence, and health care contacts. Participants were shown a book of color photographs and other descriptions of analgesics (mixed with other common drugs to blind the interviewee) marketed from the 1950s, organized by brand, name, dose, and the period during which the drugs were available to elicit an accurate lifetime history of analgesic use. Training and re-training of interviewers as well as briefing and re-briefing of study centers were standard quality control measures. Detailed logbooks were maintained in all participating dialysis centers for all new dialysis patients. The procedures to minimize potential bias included complete coverage of new dialysis patients (to address selection bias), use of index dates in the analysis (reverse causality bias), blinding of interviewers to the main research question (interviewer bias), and the restriction to phenacetin-free analgesics and adjustment for co-variables to reduce confounding. The data were collected locally, transferred to the central Data Management and Coordination Centre Berlin at the ZEG Berlin, and then to EPES Berlin for data analysis.

The SAC approved a detailed analysis plan for the "core analyses" which included a review of center performance and response rates. Frequency distributions of exposure with analgesics by case/control status and by index dates were calculated.

A fixed reference group defined as individuals who had been exposed to less than one table or unit dose of any phenacetin-free analgesic compound per month across *all *12-months periods in their lifetimes was used for all analyses. This group is comprised of all participants who indicated no or very low use of analgesics, and is called "low use" in text and tables. This strategy was adopted to account for the potential differential recall of trivial use in cases and controls, based on the notion that controls may have poorer recall of irregular and low use than cases despite all standard memory aids used (cf. protocol published in this journal [11]). The decision was approved by the SAC after discussion of intermediate analyses and became integral part of the analysis plan. All persons suspected to have ever taken phenacetin in their lifetime were excluded.

Several index dates were defined and examined to determine the most appropriate lag time between exposure to analgesics and the start of dialysis. The decision was made that exposures in the 5 years prior to start of dialysis should not be used in analyses concerning ESRD risk [11] because analgesic use may have been initiated due to incipient kidney disease during that time.

Logistic regression analyses were used to estimate the association between ESRD and analgesic exposure. Crude and adjusted odds ratios with 95% confidence interval were calculated. The decision was made a priori to use unmatched analysis if the risk estimates of matched analysis did not materially differ [11]. This is because less information is lost in unmatched analyses and these are therefore better suited for subgroup analyses.

Many possible risk factors for ESRD (cf. Table 1) were assessed for confounding. Logistic regression with backwards elimination was used to define the adjustment variables for the analytic model. The final set of adjustment variables were age (5-year group), sex (male, female), country (Germany, Austria), first degree family history of chronic renal diseases (yes, no), and self-reported exposure with welding/soldering fumes, solvents (yes, no), and education(over/under 10 years). Following the recommendation of the study's statistical advisory committee, co-variables assumed to be intermediates in the causal pathway of ESRD -such as hypertension or diabetes – were excluded from the list of possible confounders for the final analysis to avoid over-adjustment.

**Table 1:** Characteristics of ESRD cases and controls at study entry.

		Cases^1 ^N = 907		Controls^1 ^N = 3622		P
		N	%	N	%	
Age (years)	< 40	424	46.8	1644	45.4	
	40–50	483	53.2	1978	54.6	.46
Gender	Male	585	64.5	2333	64.4	
	Female	322	35.5	1289	35.6	.96
Country	Germany	726	80.0	2890	79.8	
	Austria	181	20.0	732	20.2	.87
Education (years)	- 10	603	66.5	1998	55.2	
	> 10	304	33.5	1624	44.8	< .001
Smoking	Never	340	37.5	1528	42.2	
	Current	277	30.5	694	19.2	
	Ex-smoker	290	32.0	1400	38.6	< .001
Alcohol	Seldom	757	83.5	1857	51.3	
	Often	150	16.5	1765	48.7	. < .001
						
**History of conditions, diseases**						
Diabetes mellitus	No	748	82.5	3572	98.6	
	Yes	159	17.5	50	1.4	< .001
Hypertension	No	134	14.8	3253	89.8	
	Yes	773	85.2	369	10.2	< .001
Renal diseases	No	25	2.8	3448	95.2	
	Yes	882	97.2	174	4.8	< .001
Urinary tract infections	No	802	88.4	3478	96.0	
	Yes	105	11.6	144	4.0	< .001
Family history: chronic renal disease	No	697	76.9	3425	94.6	
	Yes	210	23.1	197	5.4	< .001
Migraine	No	766	84.5	3250	89.7	
	Yes	141	15.5	372	10.3	< .001
Headache	No	656	72.3	2728	75.3	
	Yes	251	27.7	894	24.7	.06
Menstruation problems	No	233	25.7	937	25.9	
	Yes	89	9.8	352	9.7	
	n.a.	585	64.5	2333	64.4	.99
Arthritic complaints	No	828	91.3	3418	94.4	
	Yes	79	8.7	204	5.6	< .001
Chronic back pain	No	710	78.3	2866	79.1	
	Yes	197	21.7	756	20.9	.58
Psychological/neurological problems	No	829	91.4	3397	93.8	
	Yes	78	8.6	225	6.2	.01
						
**History of treatment**						
Anti-rheumatic treatment	No	892	98.4	3600	99.4	
	Yes	15	1.6	22	0.6	< .01
Immuno-suppressive therapy	No	866	95.5	3617	99.9	
	Yes	41	4.5	5	0.1	< .001
Drug treatment against cancer	No	889	98.0	3605	99.5	
	Yes	18	2.0	17	0.5	< .001
Long-term treatment with antibiotics	No	854	94.2	3526	97.4	
	Yes	53	5.8	96	2.7	.000
						
**Complaints **(subjectively compared with other persons in the same age span)						
Gastrointestinal problems	No, rare	577	63.6	2550	70.4	
	Similar/more	330	36.4	1072	29.6	< .001
Cardiovascular problems	No, rare	610	67.3	3041	84.0	
	Similar/more	297	32.7	581	16.0	< .001
Vomiting	No, rare	612	67.5	3187	88.0	
	Similar/more	295	32.5	435	12.0	< .001
Depression	No, rare	719	79.3	3172	87.6	
	Similar/more	188	20.7	450	12.4	< .001
Anxiety	No, rare	762	84.0	3259	90.0	
	Similar/more	145	16.0	363	10.0	< .001
Sleeplessness	No, rare	542	59.8	2821	77.9	
	Similar/more	365	40.2	801	22.1	< .001
Fatigue	No, rare	333	36.7	2490	68.8	
	Similar/more	574	63.3	1132	31.2	< .001
Irritability	No, rare	505	55.7	2376	65.6	
	Similar/more	402	44.3	1246	34.4	< .001
Stress	No, rare	459	50.6	1713	47.3	
	Similar/more	448	49.4	1909	52.7	< .001
Eating problems	No, rare	752	82.9	3368	93.0	
	Similar/more	155	17.1	254	7.0	< .001
						
**History of exposures at work site**						
Heavy metal	No	750	82.7	3216	88.8	
	Yes	157	17.3	406	11.2	< .001
Other metal dust	No	765	84.3	3240	89.5	
	Yes	142	15.7	382	10.5	< .001
Special silicates^2^	No	847	93.4	3431	94.7	
	Yes	60	6.6	191	5.3	.11
Other silicates^3^	No	796	87.8	3375	93.2	
	Yes	111	12.2	247	6.8	< .001
Solvents	No	751	82.8	3188	88.0	
	Yes	156	17.2	434	12.0	< .001
Welding & soldering fumes	No	797	87.9	3384	93.4	
	Yes	110	12.1	238	6.6	< .001

^1 ^Deviations from the total number of cases and controls are due to missing information

^2 ^e.g. quartz, silicon dioxide, fibre glass, silicone

^3 ^e.g. sand, cement, coal, grain dust

The analyses focused on the association between high cumulative lifetime dose (grams) and ESRD across various subgroups of analgesic formulations. The primary comparison was that of the top tertile of cumulative lifetime dose with no or very low use for users of all phenacetin-free analgesics and for users of analgesic subgroups.

Due to the small numbers in many analgesic formulation subgroups, the SAC recommended using only a minimum of adjustment variables and to refrain from calculating or reporting odds ratios if any of the cells for a comparison contained less than 10 subjects. Doses per duration of use were analyzed as well as dose-response analyses. More detailed dose-response analyses will be published at a later time in a methodology paper. Because matched and unmatched analyses showed virtually identical results, the SAC decided that this publication should show the results of unmatched analyses.

All statistical analyses were performed with the commercial statistical software Stata 8.1.

## Results

### Description of cases and controls

**Figure 1:**
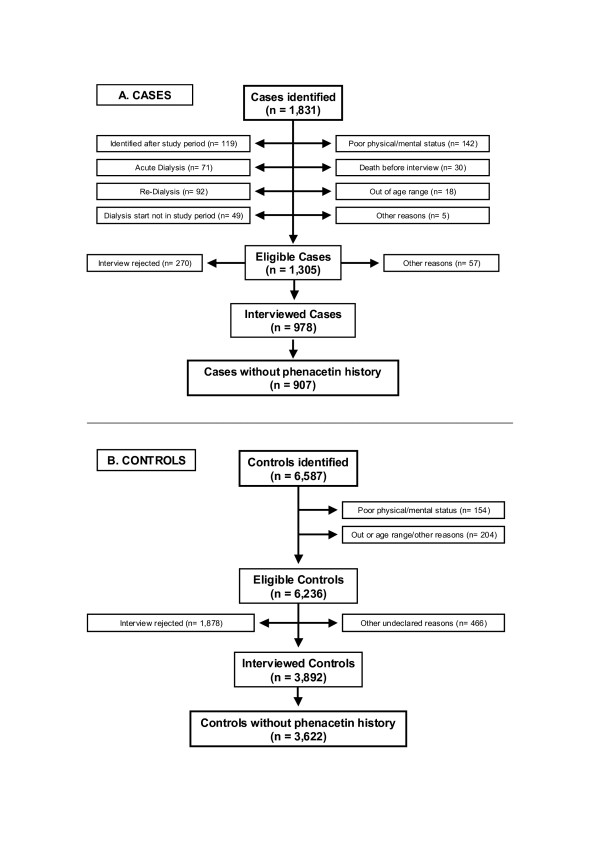
Flow chart: inclusion and exclusion of possible cases and controls.

Of 1,831 cases identified, 1,305 met the study entry criteria, 978 were interviewed, and 907 phenacetin-free cases were included in the analysis. The reasons for exclusion are shown in Figure 1. Similarly, of 6,587 potential neighborhood -controls, 6.236 were eligible, 3,892 were interviewed after 1,878 were excluded due to refusal to participate and 466 for undeclared reasons, and 3,622 controls without history of phenacetin use finally entered the analysis (Fig. 1). There were 71 cases and 270 controls with reasonable evidence for past phenacetin use mainly during childhood or early youth who were excluded from analysis. In terms of the study protocol, response rates of 74.9% for cases (978 of 1,305 eligibles) and 62.4 % (3,892 interviewed of 6,236 eligible) were considered acceptable. No important heterogeneity was observed for response rates across subgroups of age, gender, or case/control status (data not shown).

Table 1 provides an overview of baseline characteristics for all cases and their controls. As expected from the matching process, there was no difference between cases and controls with regard to age, sex, and country. Compared with cases, controls had a proportionately higher educational level (defined as more than 10 years of school), smoked less, but consumed more alcohol. Cases more frequently reported conditions suspected to play a role in ESRD development such as diabetes, hypertension, renal diseases, urinary tract infections, and a first-degree family history of chronic renal conditions. There was a higher proportion of painful conditions such as headache, migraine, chronic back or arthritic pain as well as use of specific medications (anti-rheumatic, immune-suppressive, or long-term therapy with antibiotics) among cases than among controls. Relative to their age group, controls more frequently voiced minor subjective physical and psychological complaints than did cases, which were more prone to major complaints (Table 1). Cases more often reported worksite exposure to heavy metals, other metal dust (such as aluminum, copper, chromium, tin), certain silicates (such as sand, cement, coal, rock, grain dust), solvents (such as varnish & paints, glue, crude oil, fuel, diesel, toluene), and welding and soldering fumes. These descriptive differences were taken into account for the determination of the adjustment factors for the main analysis.

The most frequently named underlying conditions leading to ESRD were glomerulonephritis (34.5%), pyelonephritis/interstitial nephritis (7.6%), polycystic kidney disease (12%), and diabetic nephropathy (15.8%), Other diseases such as lupus erythematosus, tuberculosis, vasculopathy, infarction, alport syndrome, and amyloid nephrosis were named in 18.5% of cases. The cause of kidney disease was unclear for the treating physician in 10.9%. "Analgesic nephropathy" was considered to be the cause of ESRD in only 5 of 907 cases (0.6%).

### Overall risk of ESRD, ever use, and cumulative lifetime dose

The main research question defined in the study protocol addressed the risk of ever use of phenacetin-free analgesics at index date 3 (the status 5 years before first dialysis) compared with no or low use of such substances. This standard reference group of individuals with no or very low use was defined as including those with exposure to less than one tablet or unit of any phenacetin-free analgesic compound per month across *all *12-months periods of the participant's lifetime. The adjusted odds ratio (OR) for this analysis is 0.84, 95% confidence interval (CI): 0.71 to 0.98. The crude and adjusted risk estimates were virtually identical.

The association between cumulative lifetime dose of analgesics and ESRD is shown in Table 2. The sub-categories of lifetime analgesic use are *not *mutually exclusive. The use of higher cumulative lifetime dose (tertile 3) of analgesics up to five years before dialysis was not associated with later ESRD either for all phenacetin-free analgesics together (All), or for analgesics with a single substance (monos), or for analgesics with multiple components (combis). Most risk estimates were below unity (1.0). Risk estimates calculated for medium lifetime doses (= tertile 2 and 1 combined) compared with the reference group of low use showed significantly decreased estimates. The adjusted comparison of high users of all phenacetin-free analgesics together with no or very low use showed a risk of 1.02 (95% CI: 0.81 to 1.28), the same estimate for all mono-analgesics was 0.98 (95% CI: 0.77 to 1.24). This estimate did not change when aspirin and paracetamol were excluded from the mono-preparations (OR 0.90; 95% CI: 0.61 to 1.31.). The overall estimate for the ESRD risk of high use of any combination analgesic compared with the reference group of no or very low use was 1.05 (95% CI: 0.77 to 1.44). Combination analgesics with or without paracetamol showed risk estimates in the same order of magnitude (cf. Table 2). Finally, no differences were found for the comparison between high versus low use when examining compounds with and without caffeine in this younger population with no prior use of phenacetin. We observed no evidence of an increased or different risk of ESRD associated with analgesic use (all analgesics, single substances, or combination products) with regard to the underlying disease of ESRD. The risk varied around unity across all disease subgroups containing sufficient numbers for analysis. These data will be presented at a later time in a clinical publication.

**Table 2:** Lifetime dose of analgesic use^1 ^and risk of ESRD.

	Exposure	Range grams	Cases	Controls	Adjusted OR^2 ^(95% CI)
All phenacetin-free analgesics	Low		546	2030	1.0 (referent)
	Tertile 1,2	< 217	213	1067	0.75 (0.63–0.90)
	Tertile 3	> = 217	148	525	1.02 (0.81–1.28)
**MONOS **(single substance)	Tertile 1,2	< 185	185	975	0.72 (0.59–0.87)
All monos	Tertile 3	> = 185	129	479	0.98 (0.77–1.24)
	Other		47	138	1.21 (0.84–1.74)
All- no ASA & paracetamol	Tertile 1,2	< 65	62	322	0.70 (0.51–0.94)
	Tertile 3	> = 65	43	159	0.90 (0.61–1.31)
	Other		256	1111	0.87 (0.73–1.03)
**COMBIS **(combined products)					
All combis together	Tertile 1,2	< 91	93	469	0.73 (0.56–0.93)
	Tertile 3	> = 91	75	235	1.05 (0.77–1.44)
	Other		193	888	0.84 (0.69–1.02)
All combis with paracetamol	Tertile 1,2	< 83	87	401	0,78 (0.59–1.01)
	Tertile 3	> = 83	57	197	0.95 (0.67–1.35)
	Other		217	994	0.84 (0.70–1.01)
All combis without paracetamol	Tertile 1,2	< 68	27	131	0.75 (0.49–1.16)
	Tertile 3	> = 68	26	64	1.41 (0.85–2.35)
	Other		308	1397	0.82 (0.69–0.97)
All combis with caffeine	Tertile 1,2	< 87	67	342	0.70 (0.52–0.94)
	Tertile 3	> = 87	55	169	1.01 (0.71–1.44)
	Other		239	1081	0.85 (0.71–1.02)
All combis without caffeine	Tertile 1,2	< 57	44	237	0.69 (0.49–0.99)
	Tertile 3	> = 57	37	118	1.15 (0.76–1.73)
	Other		280	1237	0.84 (0.70–0.99)

^1 ^Lifetime dose of analgesic use: medium = tertile1 and 2; high = tertile 3 vs. referent = no or very low analgesic use (exposure to less than one tablet or unit dose of any phenacetin-free analgesic compound per month across *all *12-months periods in the observed lifetime) at index date 3 (5 years before first dialysis). The category "other" means: other analgesics than those considered in the specific sub-analysis. The numbers don't add up because participants were classified into the category "other" analgesic, which is not exclusive.

^2 ^Unmatched analysis; adjusted odds ratios (OR) and 95% confidence intervals (95% CI). Adjustment for age group, sex, country, family history chronic renal diseases, exposure with welding/soldering fumes, solvents, and education

### Combined association of dose and duration

The relationship of analgesic dose and duration of use and their potential impact on ESRD development was examined extensively. Table 3 shows the analyses for all analgesics, mono-substance and combination preparations separately for lower and higher user of analgesics and for shorter and longer durations, where the cut-off points for each class are based on the respective median value found in controls. Although no significant increase is found, the table indicates that there may be a weak positive association with higher doses of analgesics used for shorter duration (left lower corner of Table 3) On the other hand, lower user of analgesics for longer durations (upper right corner) showed statistically significant lower risks for 2 of 3 analgesic types analyzed. Finally, lower use and shorter duration showed a statistically significant inverse association for all 3 analgesic types. There was an inverse association (not statistically significant) for 2 of 3 types for higher dose and longer duration of use. The numbers were too small for stable risk estimates of formulation subgroups.

**Table 3:** Dose and duration of analgesic use and risk of ESRD: All analgesics.

		**Short duration**		**Long Duration**	
		
		Cases/controls	OR (95% CI)*	Cases/controls	OR (95% CI) *
**Low dose**	All analgesics	123/580	0.78 (0.63–0.97)	36/216	0.62 (0.43–0.89)
	All monos together^1^	106/529	0.74 (0.59–0.93)	31/200	0.57 (0.39–0.85)
	All combis together^2^	49/264	0.69 (0.50–0.95)	19/84	0.84 (0.51–1.40)
**High Dose**	All analgesics	70/214	1.21 (0.91–1.61)	132/582	0.84(0.67–1.05)
	All monos together^1^	60/194	1.14 (0.84–1.55)	117/531	0.82(0.65–1.03)
	All combis together^2^	29/88	1.22 (0.79–1.88)	71/268	0.99 (0.74–1.33)

^1 ^Cases and controls that never used mono-analgesics were not shown in this table (47 cases, 138 controls for the group "all monos"

^2 ^Cases and controls that never used combination-analgesics were not shown in this table (193 cases, 888 controls for the group "all combis"

Unmatched analysis; index date 3 (5 years before start of dialysis); *adjusted *odds ratios (OR) and 95% confidence intervals (95% CI); adjusted for age, sex, country.

The cut-off point for higher/lower use and shorter/longer duration is the respective median found in controls; compared with the standard reference group with exposure to less than one tablet or unit dose of any phenacetin-free analgesic compound per month across *all *12-months periods in the observed lifetime) as referent (= 1.0): 546 cases and 2030 controls.

### Adjustment for confounding by other analgesic formulations

In order to account for overlapping of the use of different analgesic formulation during the participant's lifetime, all other analgesic subgroups were taken into the equation (Table 4). No significant association with ESRD was shown for any formulations containing ASA, paracetamol or containing neither of these two substances, i.e. the ESRD risk associated with high use (3^rd ^tertile of cumulated lifetime use) vs. low use varied around unity.

**Table 4:** Risk of ESRD and cumulative lifetime dose of Paracetamol, ASA, and substances containing neither ASA nor Paracetamol (mainly Ibuprofen) in any combination (mono- or combi) versus low use^1^

	Dose (tertiles)	Range (grams)	Cases	Controls	OR (95% CI)
No/rare use			546	2030	1.0 (referent)
ASA	I	< 50	96	431	0.96 (0.60–1.54)
	II	50–162	77	443	0.77 (0.46–1.27)
	III	163+	114	432	1.07 (0.64–1.77)
Paracetamol	I	< 17	61	297	n.d.^2^
	II	17–74	65	304	0.95 (0.63–1.42)
	III	75+	77	296	1.11 (0.74–1.67)
Other	I	< 12	38	208	0.66 (0.41–1.08)
	II	12–73	47	215	0.79 (0.50–1.23)
	III	74+	63	209	n.d.^2^

^1 ^Standard reference group with exposure to less than one tablet or unit dose of any phenacetin-free analgesic compound per month across *all *12-months periods in the observed lifetime. Unmatched analysis; Adjustment for family history, soldering fumes, solvents, education, and for age, sex, country. Adjustment for confounding by all other analgesic combinations.

^2 ^dropped due to collinearity

n.d. = no data

### Dose-response

The cumulative lifetime dose of analgesics (in grams) was stratified in eight sub-categories in steps of 500 grams up to 3.5 kg in order to show the risk distribution up to the uppermost dose groups (Table 5). There was no increased risk for ESRD up to 3.5 kg cumulative lifetime dose (98 % of the cases with ESRD). A significantly increased risk was found for the tiny subgroup of upper-most lifetime dose of more than 3.5 kg in this young age group (19 cases and 11 controls, 2% of all cases and 0.3% of all controls). Conversely, the risk was significantly reduced for the large subgroup of user with a lifetime dose up to 0.5 kg. In a second approach we examined the risk of ESRD contributed per gram at various levels of use compared with "no or very low use". This essentially confirms the above mentioned results, as each gram of use is associated with lowered risk for the group below 0.6 kg lifetime analgesics consumption, it is associated with no risk in the group up to 4.6 kg, and only in the group over 4.6 kg is there a significantly increased risk of ESRD per dose (table not shown). This threshold applies to 12 cases (= 1.3% of all cases) and 3 controls (= 0.08%) in this study. None of these were categorized as analgesic nephropathy by their treating physicians.

**Table 5:** Adjusted relative risk of ESRD by increasing cumulative lifetime dose of analgesic use (all analgesics together).

Grams	Cases	Controls	OR (95% CI)
Low use	546	2030	1.00 (referent)
-500	278	1365	0.75 (0.64–0.88)
501–1000	41	133	1.10 (0.77–1.59)
1001–1500	10	47	0.76 (0.38–1.52)
1501–2000	6	19	1.03 (0.40–2.62)
2001–2500	4	9	1.50 (0.48–4.74)
2501–3000	3	8	1.35 (0.37–4.94)
3001 +	19	11	6.02 (2.83–12.81)

Categorical modeling for dose-response analyses. Odds ratios and 95% confidence intervals. Analysis based on index date 3 (5 years before start of dialysis), adjusted for age, sex, country. ORs were not calculated if the frequency in any group was less than 10 per cell: no data (n.d.) in the table. Standard reference group of low use is defined as exposure to less than one tablet or unit dose of any phenacetin-free analgesic compound per month across *all *12-months periods in the observed lifetime.

### Description of pattern associated with uppermost cumulative lifetime use of analgesics

A detailed review of the characteristics of the 22 cases (2.4 % of all cases) and 19 controls (0.5 % of all controls) who reported at least 2.5 kg or more cumulative lifetime dose of analgesics was conducted. This cut-off point was recommended by the SAC and approved by the German Drug Authority. No difference in the reason for analgesic use for the cases and controls in this subgroup, or in their subjective complaints was determined. However, conditions associated with the causal pathway of ESRD such as hypertension, diabetes, gout, arterial diseases (cardiac, cerebral, peripheral), renal diseases, other serious diseases or operations, and family history of chronic renal diseases were much more prevalent in cases than in controls with upper-most, extreme use of analgesics. Cumulatively, 70% of the 22 cases had three and more of these seven conditions and 70% of the 19 controls had 0–1 of these conditions related to the evolution of ESRD (Table 6).

**Table 6:** Number of factors related to development of ESRD in individual cases and controls with high-end intake of analgesics (see table before).

Number of conditions^1^	Cases n = 22		Controls n = 19	
	n	%	n	%
**6**	1	4.5	0	0
**5**	4	18.2	0	0
**4**	6	27.3	0	0
**3**	5	22.7	0	0
**2**	5	22.7	6	31.6
**1**	1	4.5	7	36.8
**0**	0	0	6	31.6

^1 ^The following seven conditions were considered for this table: Arterial problems (coronary, brain, peripheral), hypertension, diabetes mellitus, gout, renal diseases, other serious diseases/operations, and family history of chronic renal diseases (first degree relatives)

## Discussion

The objective of this study was the investigation of the effect of lifetime use of phenacetin-free analgesics on ESRD occurrence in a younger population. The restriction to a study group below the age of 50 was a prerequisite for the effective exclusion of phenacetin use, and we believe that this is the first study to completely avoid phenacetin contamination in the assessment of ESRD associated with analgesic uses. The results suggest that there is no association between ESRD and analgesic use in general, nor with the use of specific analgesics or combinations with or without additional caffeine in the age group below 50 years.

The direct comparison of baseline users of no or very low lifetime analgesic doses with the tertile distributions of lifetime doses in grams of the user group shows no increased ESRD risk associated with phenacetin-free analgesics in the highest tertile. Instead, there was a significantly reduced risk ESRD for the lower two tertiles for all analgesics, mono-preparations, and combination products. No clinically relevant increase of ESRD risk with increasing dose was found in an analysis of the effect of ASA, paracetamol, and other ingredients with full adjustment including other analgesic combinations. Users of lower doses of analgesics showed a decreased risk of ESRD independent of duration of use (Table 3). The group of users of high lifetime doses showed a non-significant tendency for higher risks when these substances were taken within a shorter period. We cannot draw any strong conclusions from the findings related to the combined associations of dose and duration of analgesic use.

The detailed examination of dose-response distributions showed no association or significantly reduced risks of ESRD for almost all levels of analgesic use. However, a tiny group of cases and controls showed a significantly increased risk associated with a dose over 3.5 kg in the dose-stratified analyses. This was found to significantly increase around a dose of 4–5 kg lifetime use of analgesics in the continuous dose analyses. This level of use corresponds to a daily consumption of 200 to 400 grams of analgesic over a 30-year period. Because the numbers in this subgroup were too low for estimation, a detailed description was prepared for this upper-most dose group (22 cases and 19 controls). The main distinction between cases and controls were the much higher prevalence of factors closely associated with later development of ESRD among the cases, so that we interpret this group as being defined by their high-risk status rather than by their use of analgesics which is incidental to their prior health condition. The same conclusion was reached by an external expert in nephrology (Prof. Michielsen) who was asked by the Scientific Advisory Committee of the study and by the German Drug Authority to individually evaluate the likelihood that high analgesic use in these cases is is the cause of their ESRD. Prof. Michielsen expressed in his report that the individual assessment of lifetime users of 2.5 kg analgesic user indicated:1)There is no evidence of classical analgesic nephropathy, and 2) that there is no indication that analgesic use influenced the evolution to ESRD in this small group of extremely high users (cf. full assessment report at the SAN website [12]).

### Strengths and weaknesses of the study

The two important advantages of this study are the use of a 5-year lag-time across all analyses on analgesic exposure and ESRD risk and the presumably complete exclusion of past use of phenacetin. The 5-year lag time has previously been used in only two [14,15] studies. It addresses the issue of temporality and reduces the risk of showing estimates based on analgesic use prompted by an incipient renal condition rather than on the occurrence of a renal condition following a period of analgesic use (reverse causality bias). To our knowledge, no previous study has eliminated phenacetin use from its population to the extent this study has, and we consider this a condition sine qua non for the estimation of ESRD risk associated with the currently available analgesics.

Every effort was made to minimize recall and information bias by using face-to-face interviews with a set of aids and procedures to jog memory, check the plausibility of recalled analgesic use, and to provide similar conditions for cases and controls. Selection bias is addressed in the community-based approach of this case-control study. Logbooks were maintained in dialysis centers to assure complete coverage. Nonetheless, and although comparable with other studies in this area, a non-response rate of around 30% leaves some possibility for bias. It is reassuring that the information available on non-responders did not differ from the information for those included in the study. Finally, the study suffers the limitations of all observational studies in that the participation of cases may be very much determined by intentions of the treating physician who invited patients, while the situation could differ for controls. Differences in reporting behavior between cases and controls can never be entirely excluded, and controls who consumed large amounts of analgesics may have a poor health-related quality of life which prevents them from volunteering for such a study.

The age limit of 50 years is strength of the study because it largely eliminates phenacetin users from this population, but it may also restrict the generalizability of the findings. However, this study feature was considered an essential safeguard against confounding by phenacetin use. Reliable results for the risk of ESRD and analgesic use in the population aged 50 to 70 years, for example, could be obtained only in the decades when phenacetin users had slowly depleted. Even then, the much higher potential of serious confounding and bias in the higher age group would make it difficult to interpret an association because, if any association is found, it is likely to be small. Although the possibility that some persons might not have sufficient time to accumulate a harmful lifetime dose up to the age of 50 years, we consider the evidence of no clinically important association between analgesic use and ESRD provided by this study to be the best currently available. Therefore, and in the absence of other reliable evidence, the results of this study most likely also apply to higher age groups.

The debate on analgesic use and nephropathy has gone on for decades and is not yet concluded [16]. Many earlier studies which did not exclude past phenacetin use found a significant association between analgesic use and chronic renal failure/ESRD with relative risk increases ranging from 2- to 8-fold [17-22]. Other studies observed no or no clearly increased risk or variations among formulations [14,15,23,24] or else were inconclusive [25,26]. Fored *et al.* (2003) [15] did not rule out that the associations found in their study might be due to bias, i.e. that the use of analgesics was triggered by conditions associated with the renal disease.

Recently, Mihatsch *et al.* (2006) [27], conducted an exact replication of the benchmark autopsy studies on analgesic nephropathy which he performed 1978–80 in Basel [28]. The author wrote: "The last and single autopsy case of proven classic analgesic nephropathy detected only with the present sophisticated histological study at the end of the year 2000 can thus be taken as further evidence that this type of chronic renal disease was due to nothing but phenacetin-containing mixed analgesics. Some 20 years after removal of phenacetin from the market classic analgesic nephropathy is all but disappearing and will no longer be a health hazard in the 21^st ^century." [27]. The results of the present study on a population of individuals with no prior phenacetin use supports this finding.

The present study adds to the available data characterizing the association of ESRD risk and lifetime consumption of analgesics applying advanced methods. Our data show no significant association between ESRD and analgesic use in general or for the use of specific analgesics or combinations with or without additional caffeine. The descriptive analysis of a small highly exposed subgroup indicates that their very high cumulative lifetime use of analgesics is based on a pattern of chronic, painful ill health and does not constitute a causal association with ESRD. However, further research on extreme users of analgesics is recommended.

## Conclusion

We found no clinically meaningful evidence for an increased risk of ESRD associated with use of phenacetin-free analgesics in single or combined formulation. The apparent risk increase shown in a small subgroup with extreme lifetime dose of analgesics is most likely an indirect, non-causal association. This hypothesis, however, cannot be confirmed or refuted within our case-control study. Overall, our results lend support to the mounting evidence that phenacetin-free analgesics do not induce ESRD and that the notion of "analgesic nephropathy" needs to be re-evaluated.

## Competing interests

Investigators, drug regulators and the pharmaceutical companies in a common approach initiated this study. The investigators and the SAC exclusively designed the study with some critical remarks from drug regulators and industry. The SAN study was scientifically independent and governed by an independent Advisory Committee (SAC). A group of pharmaceutical companies (Boehringer-Ingelheim Pharma GmbH&Co.KG; Dr.Mann Pharma; Whitehall-Much GmbH; Berlin-Chemie AG; Dr. R. Pfleger GmbH; Roche Consumer Health) provided an unconditional grant to cover the costs of the preparatory meetings, the conduct of the study, and the meetings of the advisory committees. The investigators were accountable to the SAC in all scientific matters. None of the investigators have any financial relationship with the group of manufacturers of analgesics.

## Authors' contributions

FvdW: Responsible for the nephrological issues of the study protocol, the collaboration of German nephrological societies and dialysis centres and contributed to writing/revising of the paper. Prof. van der Woude died in December 2006. LAJH: Responsible for the epidemiological study design, evaluation plan, and involved in writing of the paper. HG: Responsible for the nephrological issues, the collaboration of Austrian nephrological society and dialysis centres, and contributed to writing/revising of the paper. ML: Contributed to the data management & study evaluation plan and oversaw the data processing and contributed to revising the manuscript. SM: Contributed to the planning of the international field work and particularly data management, development of questionnaires and revisions of the manuscript. AA: Leading activities during the development of all questionnaires and forms, responsible for training and quality assurance of the field work across centres, contributed to revising the manuscript. DKH: responsible for the statistical evaluation of the study, contributed to revisions of the manuscript.

All authors read and approved the final manuscript-

## Pre-publication history

The pre-publication history for this paper can be accessed here:


